# Supplementary material for: Three-dimensional mapping reveals heterochronic development of the neuromuscular system in postnatal mouse skeletal muscles
Source: Commun Biol. 2022 Nov 8;5:1200. doi: 10.1038/s42003-022-04159-1 (PMC9643545; doi:10.1038/s42003-022-04159-1)
Supplement: Supplementary file 2 — Supplementary Information [file 42003_2022_4159_MOESM2_ESM.pdf]

Supplementary Information

Three-Dimensional Mapping Reveals Heterochronic Development of the Neuromuscular System in Postnatal Mouse Skeletal Muscles

Jianyi Xu[1,2†], Jingtan Zhu[1,2†], Yusha Li[1,2], Yingtao Yao[1,2], Ang Xuan[1,2], Dongyu Li[1,2], Tingting Yu[1,2*], Dan Zhu[1,2*]

[1]Britton Chance Center for Biomedical Photonics - MoE Key Laboratory for Biomedical Photonics, Wuhan National Laboratory for Optoelectronics - Advanced Biomedical Imaging Facility, Huazhong University of Science and Technology, Wuhan, Hubei 430074, China.

[2]Optics Valley Laboratory, Hubei 430074, China.

*Correspondence: yutingting@hust.edu.cn, dawnzh@mail.hust.edu.cn

†These authors contributed equally to this work.

Supplementary Figure 1: Postnatal development of myofibers in the tibialis anterior.

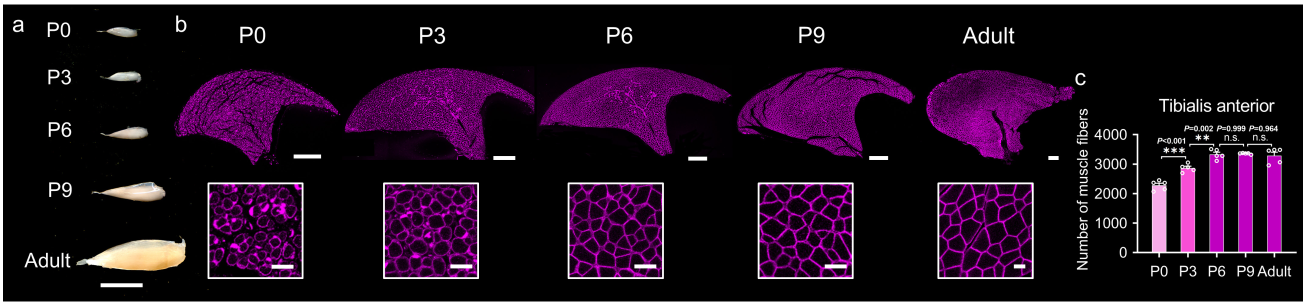

(a) The tibialis anterior at different time points (P0, P3, P6, P9, and adulthood). Scale bar: 5mm. **(b)** Fluorescence imaging of myofiber membranes immunostained with the anti-Dystrophin antibody at the cross-section of the muscle belly in developing and adult tibialis anterior; scale bar: 200 μm. Images in the white frames show the detailed morphologies of myofibers; scale bar: 25 μm. **(c)** The quantification of myofiber numbers in (b) (n=5 independent animals for each time point). All values are presented as the means ± SEM; statistical significance (n.s. represents not significant, and $**P < 0.01$, $***P < 0.001$) was assessed using one-way ANOVA followed by Tukey post hoc test.

Supplementary Figure 2: 3D imaging and analysis of the innervation pattern in the tibialis anterior during postnatal development.

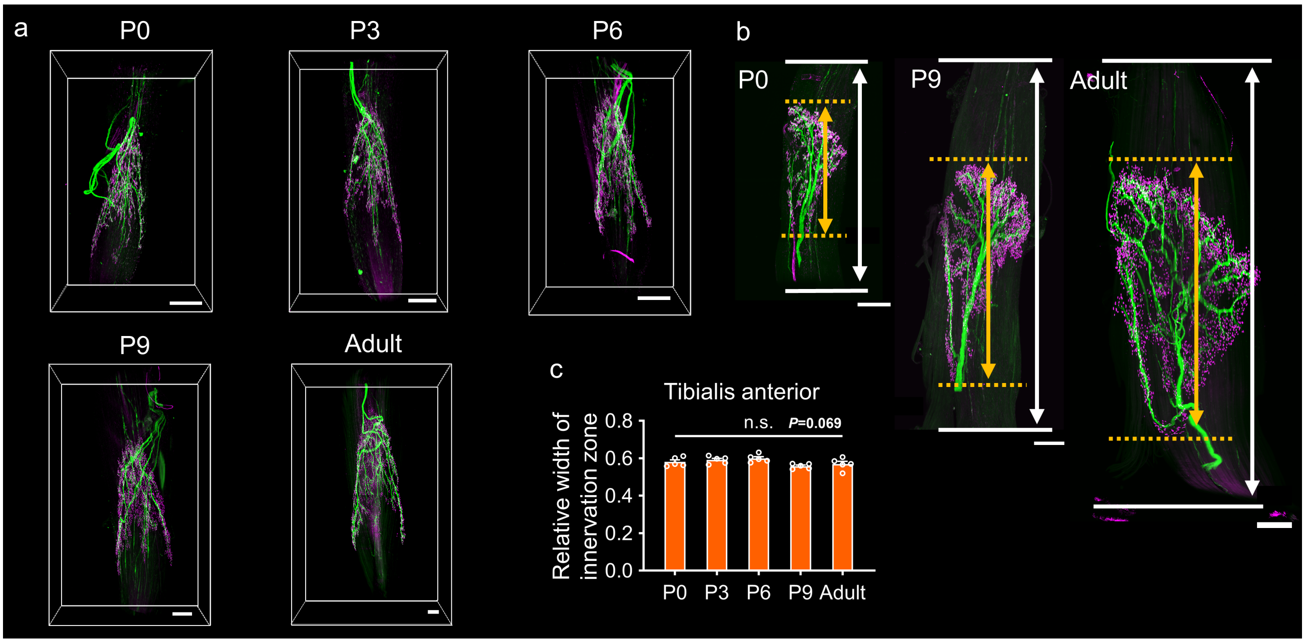

(a) 3D reconstructions of intramuscular motor nerves and NMJs in the tibialis anterior during postnatal development and adulthood. **(b)** Fluorescence imaging showing how the relative width of the innervation pattern of the biceps brachii was measured at P0, P9, and in adults. The yellow arrows indicate the widths of the innervation pattern, and the white arrows represent the widths of whole muscles. **(c)** The quantification of the relative range of the innervation zone in whole muscles (n=5 independent animals for each time point). All values are presented as the means ± SEM; statistical significance in (n.s. represents not significant) was assessed using one-way ANOVA. Scale bar: 300 μm.

Supplementary Figure 3: Comparison between intramuscular innervation patterns in the cleidomastoid during postnatal development.

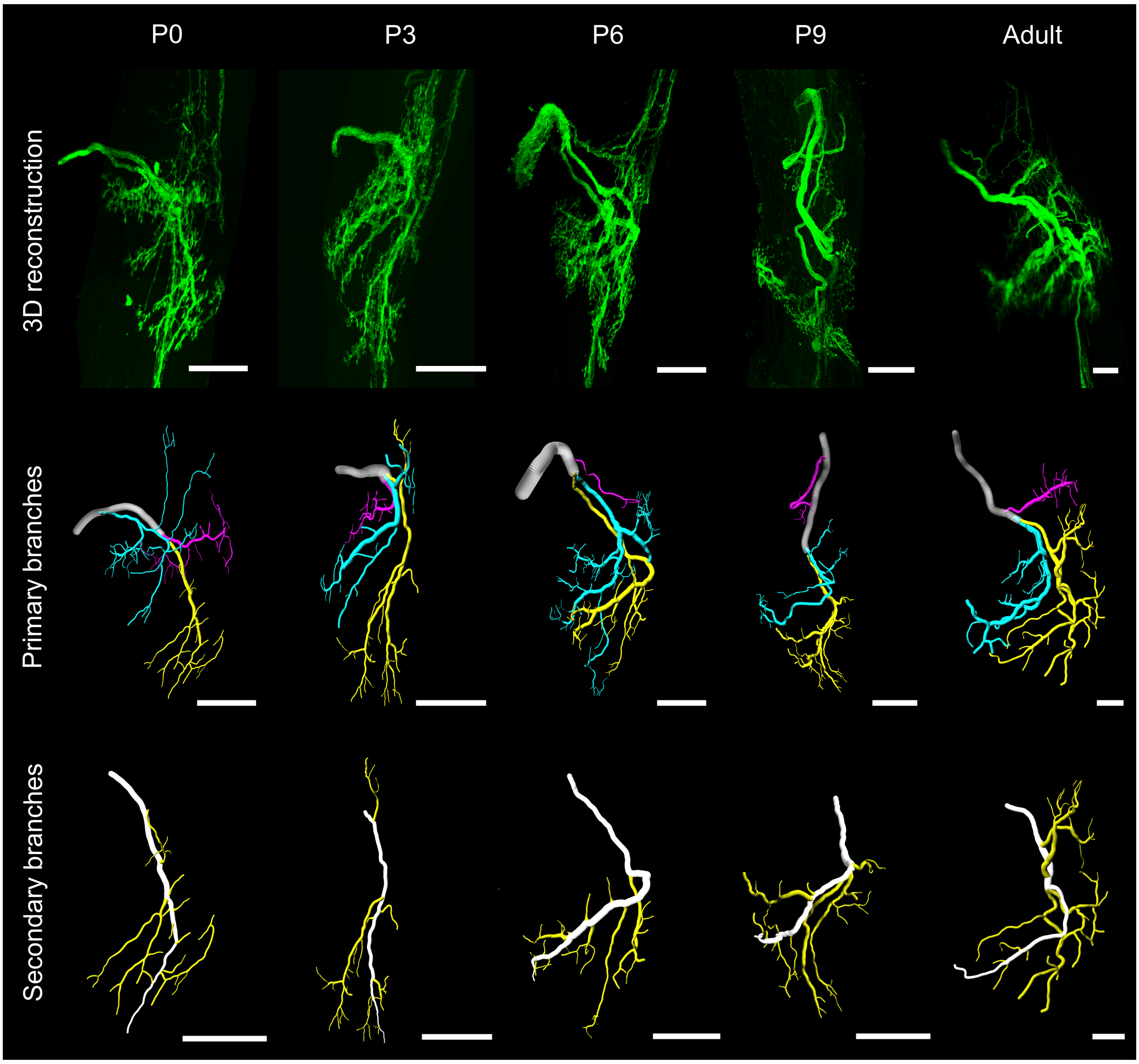

The segmentation of nerve branches by 3D tracing using the Imaris software is shown in the second row. Primary branches are marked with different colors. The yellow primary branches at different time points were selected for further analysis of secondary branches, as shown in the third row. Scale bar: 200 µm.

Supplementary Figure 4: Comparison between intramuscular innervation patterns in the biceps brachii during postnatal development.

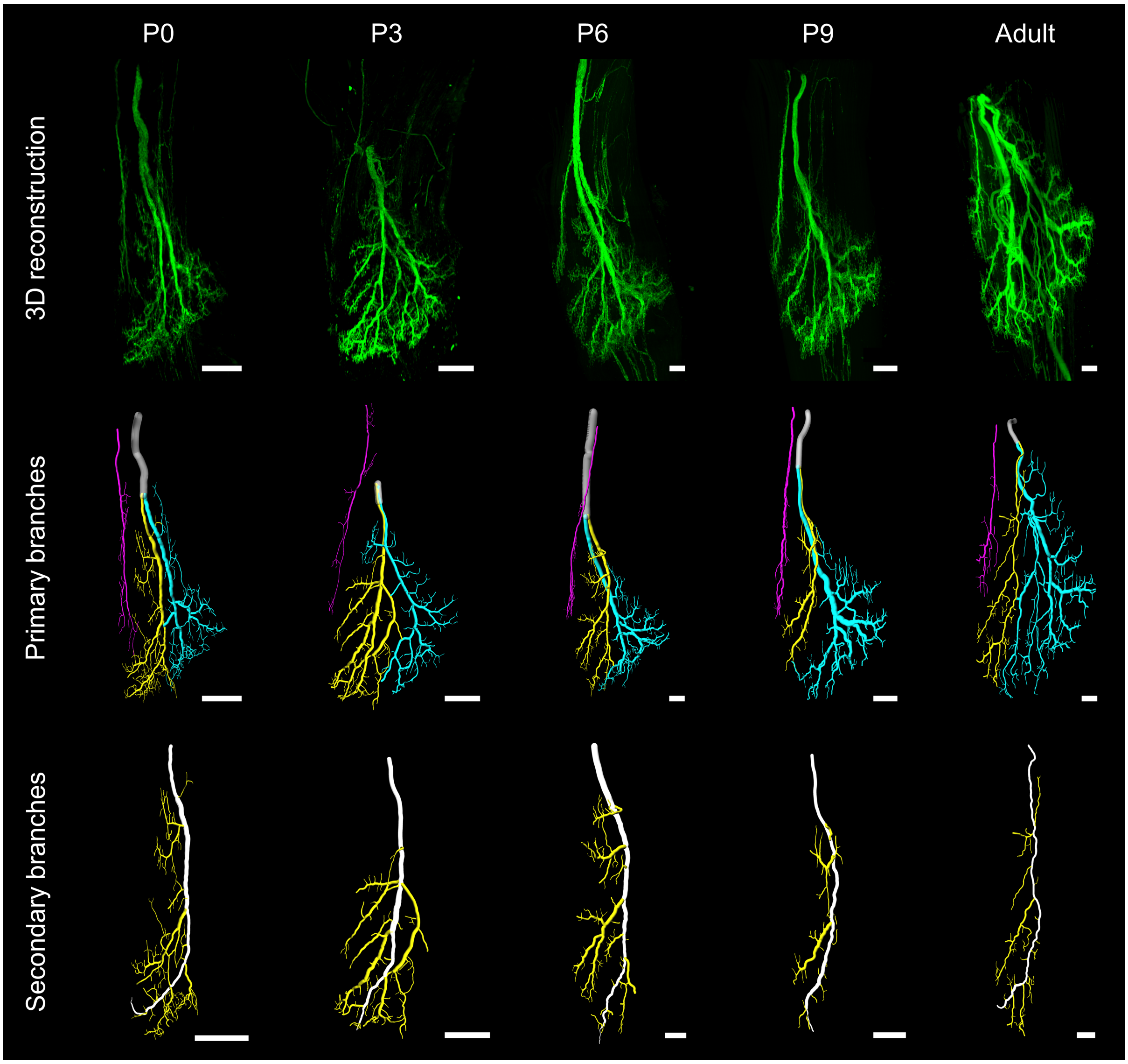

The segmentation of nerve branches by 3D tracing using the Imaris software is shown in the second row. Primary branches are marked with different colors. The yellow primary branches at different time points were selected for further analysis of secondary branches, as shown in the third row. Scale bar: 200 μm.

Supplementary Figure 5: Comparison between intramuscular innervation patterns in the gastrocnemius during postnatal development.

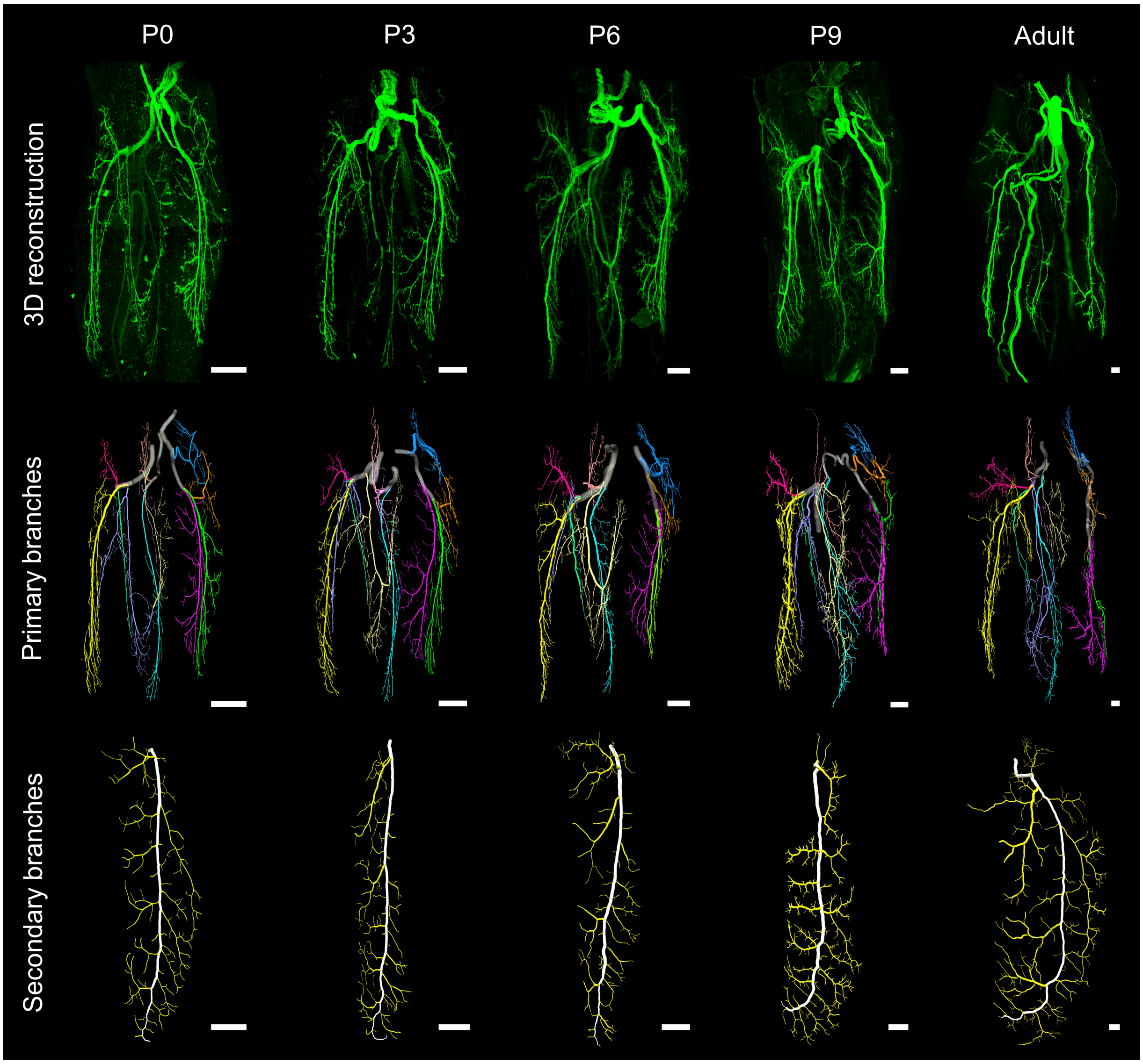

The segmentation of nerve branches by 3D tracing using the Imaris software is shown in the second row. Primary branches are marked with different colors. The yellow primary branches at different time points were selected for further analysis of secondary branches, as shown in the third row. Scale bar: 200 μm.

Supplementary Figure 6. 3D analysis of NMJs in different skeletal muscles during postnatal development.

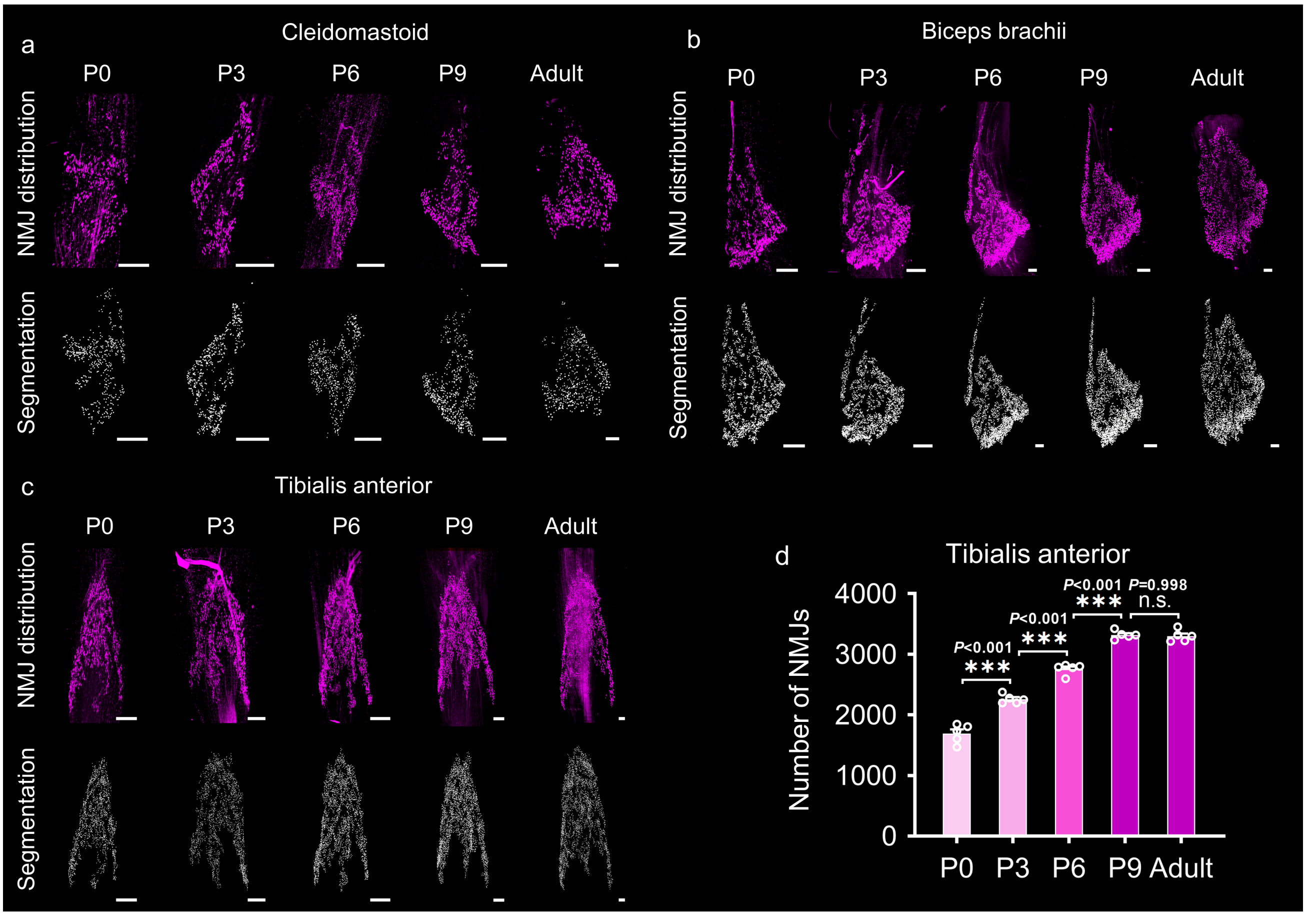

3D distribution and segmentation of NMJs in the cleidomastoid **(a)**, biceps brachii **(b)**, and tibialis anterior **(c)** at different time points (P0, P3, P6, P9, and adulthood). **(d)** The quantification of NMJs in the tibialis anterior at different time points (n=5 independent animals at each time point). Scale bar: 200 μm. All values are presented as the means ± SEM; statistical significance (n.s. represents not significant and ****P* < 0.001) was assessed using one-way ANOVA followed by Tukey post hoc test.

Supplementary Figure 7: The procedure for calculating myofiber numbers.

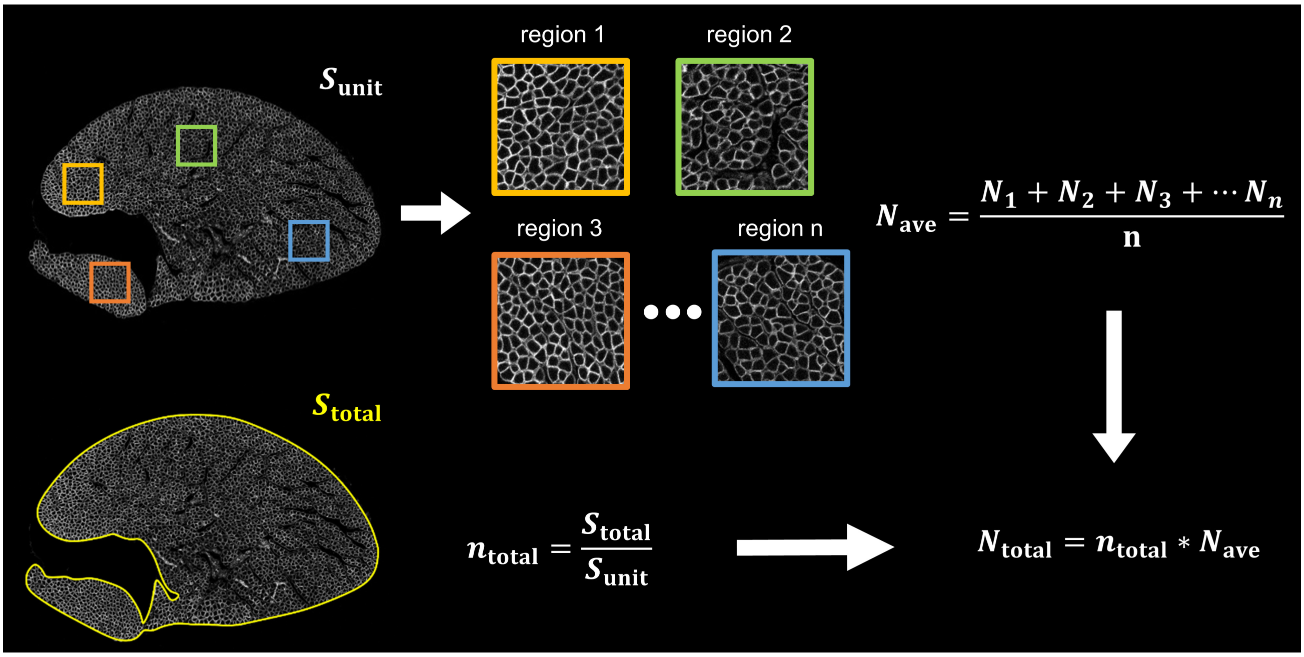

The "Rectangle" tool in the Imaris was used to crop several regions (n) with equal areas (S_{unit}) as unit regions. The fiber numbers in these unit regions were counted (N_1, N_2, N_3, …, and N_n), and the averages (N_{ave}) were calculated. The "Freehand Selection" tool was used to determine the edge of whole muscle sections, and the total area was measured as S_{total}. Subsequently, the number of unit regions contained in whole muscle sections was calculated and denoted as n_{total}. Finally, the total number of myofibers (N_{total}) in whole sections was calculated by multiplying n_{total} by N_{ave}.

Supplementary Figure 8: Quantification of the innervation pattern using Filament module in the Imaris.

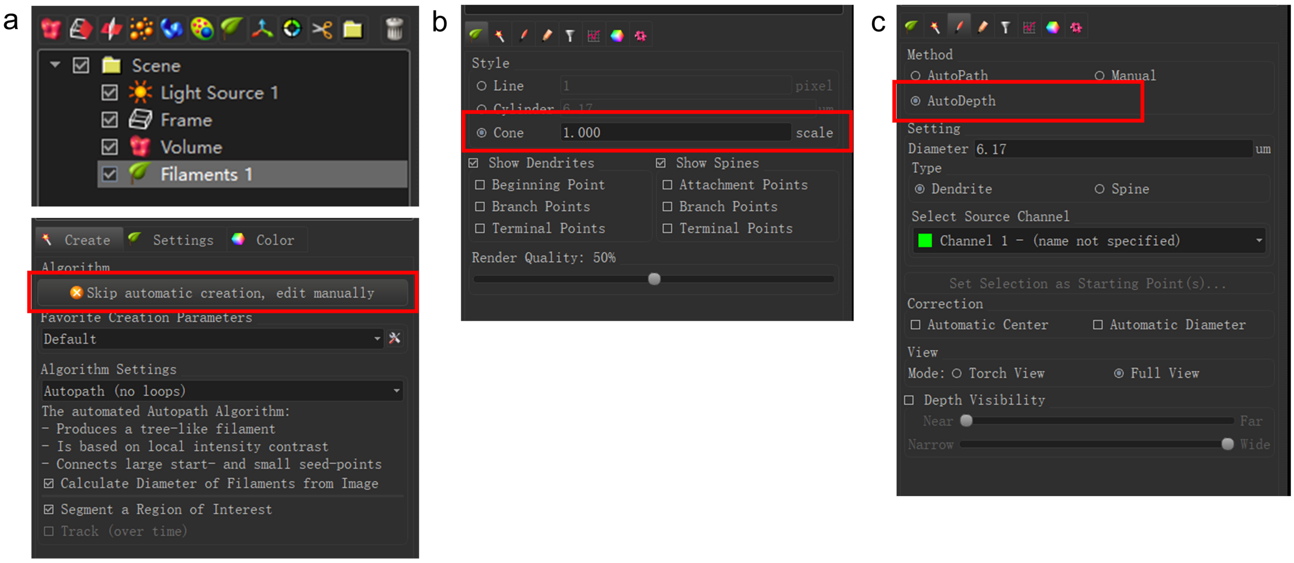

(a) Select the Filament module in the toolbar and skip automatic creation. **(b)** Select the “Cone” style. **(c)** Select “AutoDepth” style.

Supplementary Figure 9: Quantification of NMJ number using Spots module in the Imaris.

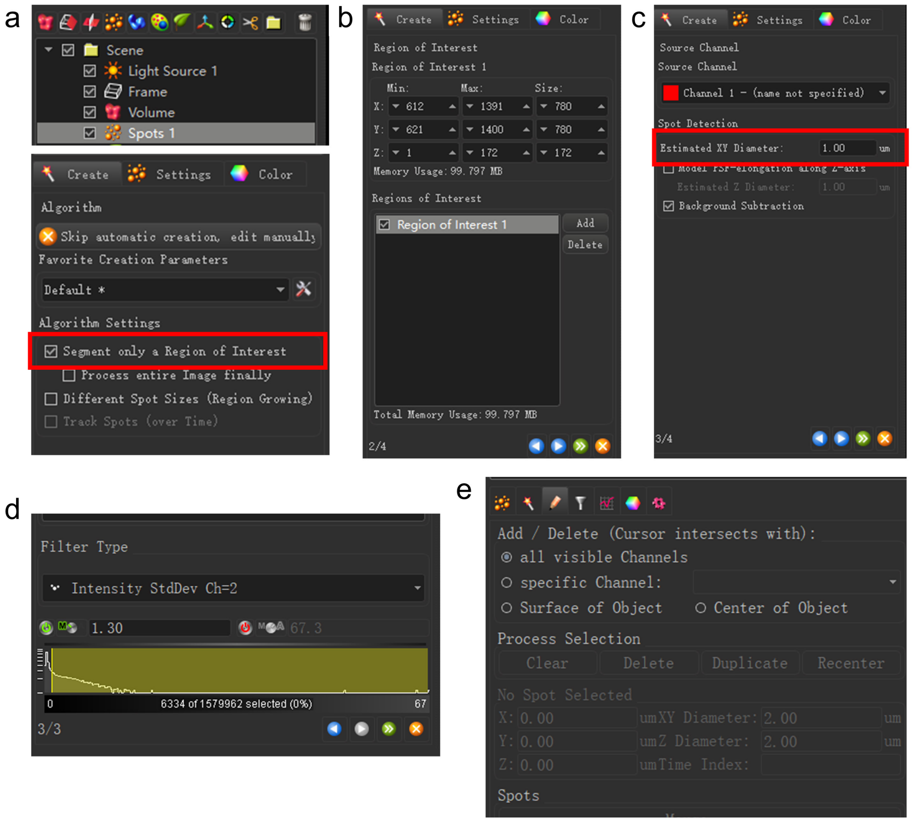

(a) Select Spot module and check the calculation mode. **(b)** Set the size of calculated region. **(c)** Enter the diameter of particles. **(d)** Select the “Intensity StdDev” algorithm and adjust the threshold range. **(e)** Select the “Add/Delete” mode to correct results using manual selection.
