# Supplementary material for: Three-dimensional mapping reveals heterochronic development of the neuromuscular system in postnatal mouse skeletal muscles
Source: Commun Biol. 2022 Nov 8;5:1200. doi: 10.1038/s42003-022-04159-1 (PMC9643545; doi:10.1038/s42003-022-04159-1)
Supplement: Supplementary file 3 — Description of Additional Supplementary Files [file 42003_2022_4159_MOESM3_ESM.pdf]

Description of Additional Supplementary Files

File name: Supplementary Data 1

Description: source data underlying Fig 1a-b, 1d, 1f, 1h, 2c, 2e, 2g-i, 3e-g, 4b-c, 5j-l, 6b-d.

File name: Supplementary Movie 1

Description: 3D visualization of innervating nerves in the cleidomastoid during postnatal development.

File name: Supplementary Movie 2

Description: 3D visualization of innervating nerves in the biceps brachii during postnatal development.

File name: Supplementary Movie 3

Description: 3D visualization of innervating nerves in the gastrocnemius during postnatal development.
